# High-performance liquid chromatography with fluorescence detection for mycophenolic acid determination in saliva samples

**DOI:** 10.1007/s43440-023-00474-4

**Published:** 2023-03-11

**Authors:** Joanna Sobiak, Matylda Resztak, Joanna Banasiak, Jacek Zachwieja, Danuta Ostalska-Nowicka

**Affiliations:** 1 Department of Physical Pharmacy and Pharmacokinetics, Poznan University of Medical Sciences, 3 Rokietnicka Street, 60-806 Poznan, Poland; 2 Department of Pediatric Nephrology and Hypertension, Poznan University of Medical Sciences, Poznan, Poland

**Keywords:** Mycophenolate mofetil, Therapeutic drug monitoring, HPLC, Saliva, Nephrotic syndrome, Mycophenolic acid

## Abstract

**Background:**

For therapeutic drug monitoring (TDM) of mycophenolic acid (MPA), which is frequently proposed, saliva might be a suitable and easy-to-obtain biological matrix. The study aimed to validate an HPLC method with fluorescence detection for determining mycophenolic acid in saliva (sMPA) in children with nephrotic syndrome.

**Methods:**

The mobile phase was composed of methanol and tetrabutylammonium bromide with disodium hydrogen phosphate (pH 8.5) at a 48:52 ratio. To prepare the saliva samples, 100 µL of saliva, 50 µL of calibration standards, and 50 µL of levofloxacin (used as an internal standard) were mixed and evaporated to dryness at 45 °C for 2 h. The resulting dry extract was reconstituted in the mobile phase and injected into the HPLC system after centrifugation. Saliva samples from study participants were collected using Salivette^®^ devices.

**Results:**

The method was linear within the range of 5–2000 ng/mL, was selective with no carry-over effect and met the acceptance criteria for within-run and between-run accuracy and precision. Saliva samples can be stored for up to 2 h at room temperature, for up to 4 h at 4 °C, and for up to 6 months at − 80 °C. MPA was stable in saliva after three freeze–thaw cycles, in dry extract for 20 h at 4 °C, and for 4 h in the autosampler at room temperature. MPA recovery from Salivette^®^ cotton swabs was within the range of 94–105%. The sMPA concentrations in the two children with nephrotic syndrome who were treated with mycophenolate mofetil were within 5–112 ng/mL.

**Conclusions:**

The sMPA determination method is specific, selective, and meets the validation requirements for analytic methods. It may be used in children with nephrotic syndrome; however further studies are required to investigate focusing on sMPA and the correlation between sMPA and total MPA and its possible contribution to MPA TDM is required.

**Graphical abstract:**

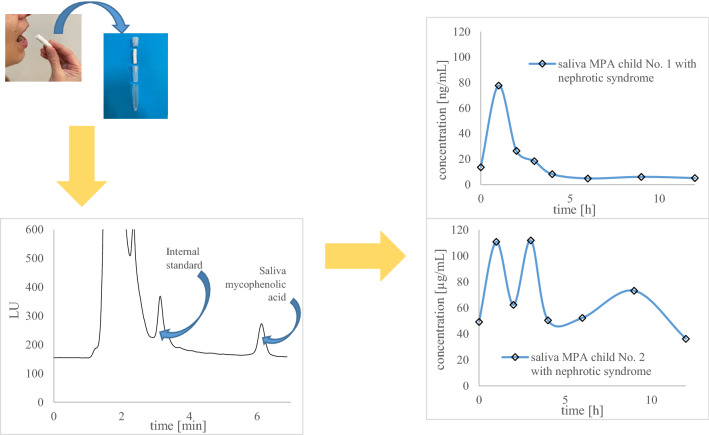

## Introduction

Dose adjustment of mycophenolic acid (MPA) is a recommended standard of practice in various conditions including solid organ transplantation [1]. However, the optimal method for therapeutic drug monitoring (TDM) of MPA is still being discussed, particularly in other indications such as autoimmune diseases and pediatric nephrotic syndrome, which are different from the original indications of acute rejection prophylaxis after solid organ transplantation [1, 2]. Furthermore, in children with nephrotic syndrome, higher target values of pharmacokinetic parameters have been suggested [3–5]. MPA is highly protein bound (> 98%) [6, 7], and there is still debate regarding whether total MPA (tMPA) or free MPA (fMPA), which is unbound to plasma protein, correlates better with the drug’s pharmacological effect [1]. However, measuring unbound plasma levels has the disadvantages of invasive blood sampling and additional filtration steps [8]. Therefore, alternative TDM approaches are still needed such as a limited sampling strategy (LSS) for estimating MPA area under the concentration time from 0 to 12 h curve (AUC_0–12_) [9–11]. Other approaches have focused on pharmacodynamic or pharmacogenetic biomarkers, as well as the use of matrices other than plasma for MPA determination [1, 2, 12].

Oral fluid is one of the matrices that has numerous advantages and could be useful in TDM. TDM based on saliva is a non-invasive, convenient, easy, and rapid procedure that may be applied to different patient groups regardless of age [13, 14]. It is postulated that saliva concentrations reflect the unbound drug levels, which is the pharmacologically active form of plasma MPA [8, 15]. Hence, using saliva as an alternative biological fluid for drug concentration measurement could offer a more acceptable approach [16]. However, TDM based on saliva as a matrix may be limited by small specimen volumes, low analyte concentrations, interference from food particles or drugs, changes in pH, drug concentration with saliva flow stimulation, and the requirement for standardized testing procedures [15, 17].

MPA can be classified as a class III compound in the Salivary Excretion Classification System (SECS III), which means it exhibits high permeability and high binding in plasma, and can potentially be excreted in saliva [8, 18]. To date, several studies have confirmed that MPA is secreted in saliva and have shown a good correlation between saliva and plasma MPA exposures [8, 16, 19, 20]. However, more recently, two studies have reported contradictory results [15, 21]. All studies on salivary MPA (sMPA) have employed liquid chromatography tandem mass spectrometry (LC–MS/MS) for sMPA determination [8, 15, 16, 19, 20, 22], but none have used high-performance liquid chromatography with fluorescence detection (HPLC-FLD). The HPLC-FLD method may have some advantages, such as lower analysis cost and greater availability of HPLC equipment than LC–MS/MS. Additionally, none of these studies have included children with nephrotic syndrome.

The aim of this study was to validate the HPLC-FLD method for MPA determination in saliva samples collected from children with nephrotic syndrome who were being treated with mycophenolate mofetil (MMF), a prodrug of MPA. To the best of our knowledge, this is the first study to utilize the HPLC-FLD method for MPA determination in saliva. Our study was based on the HPLC-FLD method for determining free MPA (fMPA) in plasma samples [3, 23].

## Material and methods

### Materials

All solvents and reagents used in this study were of HPLC or analytical grade. MPA (Product No. M5255) and levofloxacin (internal standard, IS) (Product No. 28266) were purchased from Sigma-Aldrich (Germany). Methanol (Product No. 106007) and acetonitrile (Product No. 100030) were obtained from Merck. Tetrabutylammonium bromide (TBAB; Product No. 193119) and disodium hydrogen phosphate (Product No. S0876) (both from Sigma-Aldrich, Germany) were used to prepare the mobile phase. Demineralized water was used throughout (Simplicity UV, Millipore, USA). Drug-free saliva samples were obtained from healthy volunteers (Decision number 773/21).

### Chromatographic conditions and apparatus

The analysis was performed using the HPLC system HP1100 (Hewlett Packard, Austria). The mobile phase consisted of methanol and 15 mM TBAB with 10 mM disodium hydrogen phosphate buffer adjusted to pH 8.5, mixed at the ratio of 48:52 (v/v) and degassed in an ultrasonic bath. The analysis was conducted at a flow rate of 1.0 mL/min, and the temperature of the chromatographic separation was set to 40 °C. The analytical column used for sMPA determination was Zorbax Eclipse XDB C18, 150 mm × 4.6 mm, 5 µm, protected by a guard column Eclipse XDB-C18 4.6 mm × 12.5 mm, 5 µm, both from Agilent (USA). Fluorescence detection (FLD) was used with an excitation wavelength of 324 nm and an emission wavelength of 425 nm. The HPLC system was controlled by ChemStation software.

### Calibration curves

Stock solutions of 1.0 mg/mL of MPA and 1.0 mg/mL of IS were prepared by dissolving the appropriate amount of MPA or IS in methanol. Calibration standards were then prepared by diluting stock solutions. Specifically, the following MPA calibration standards in methanol were prepared: 10, 20, 40, 100, 200, 400, 2000, and 4000 ng/mL. The IS standard solution had a concentration of 2000 ng/mL in acetonitrile. To prepare samples for analysis, 100 µL of blank saliva was mixed with 50 µL of the appropriate calibration standard and 50 µL of IS solution, resulting in MPA concentrations in the matrix of 5, 10, 20, 50, 100, 200, 1000, and 2000 ng/mL, and an IS concentration of 1000 ng/mL. The samples were then evaporated to dryness at 45 °C in a centrifugal vacuum concentrator (Eppendorf, Germany) and the resulting dry extract was reconstituted in 100 µL of mobile phase. After double centrifugation for 5 min at 14,000×*g*, 20 µL was injected into HPLC system.

The concentrations of tMPA and fMPA were determined in the collected plasma samples. The HPLC with ultraviolet (UV) detection [3, 24] and HPLC-FLD [3, 23] methods were applied for the determination of tMPA and fMPA, respectively. For both methods, the analytical column used was Zorbax Eclipse XDB C18 (150 mm × 4.6 mm, 5 µm, Agilent Inc., USA). The calibration curves were linear within the range 0.250–40.0 µg/mL and 2.5–1000 ng/mL for tMPA and fMPA, respectively. Phenytoin dissolved in 0.1 mol/L orthophosphoric acid in acetonitrile was used as an IS for tMPA, and the mobile phase consisted of methanol and 0.15% phosphoric acid. The UV detection was at 215 nm [3, 24]. To obtain fMPA plasma fraction, Amicon Ultra 0.5 Centrifugal Filter Units with a molecular weight cutoff of 10 kD were used. Plasma samples were centrifuged at 14,000*g* for 30 min at 25 °C. For fMPA, the mobile phase consisted of a buffer (10 mmol/L disodium hydrogen phosphate, 15 mmol/L of TBAB, pH 8.5) and methanol (40:60, v/v). No IS was used in this method. For FLD, the excitation was at 342 nm, and the emission was at 425 nm [3, 23].

### Method validation

The developed method was validated in accordance with the guidelines for bioanalytical method validation issued by the European Medicines Agency [25].

Selectivity was assessed in eight different samples of blank saliva obtained from healthy adult volunteers. This was done by comparing the chromatogram of blank saliva, which had been processed by evaporating to a dry extract, with the chromatogram of saliva spiked with MPA and IS to detect any interfering peaks. A few potentially co-administered drugs, such as paracetamol, voriconazole, itraconazole, ketoconazole and amlodipine, were also examined to exclude possible interferences with the target compound or IS.

The lower limit of quantification (LLOQ) was defined as the lowest concentration of MPA that can be determined by the method within ± 20% of the nominal concentration. Additionally, the analyte signal of the LLOQ sample was required to be at least 5 times the signal of a blank sample, as per the guidelines [25].

The calibration curve was constructed by plotting the ratio of peak area of sMPA to IS against the nominal concentration of sMPA. The sMPA concentrations in matrix ranged from 5 to 2000 ng/mL. Linearity was evaluated using Student’s test-test and the correlation coefficient (r) was calculated.

To assess accuracy and precision, within-run and between-run measurements were performed on the LLOQ, low (10 ng/mL), medium (1000 ng/mL), and high (2000 ng/mL) quality control (QC) samples with 5 replicates for each. Accuracy was reported as the relative error (in %) and was calculated as the difference between the mean concentration determined and the nominal value. Precision was expressed as the coefficient of variation (CV%).

Carry-over was evaluated by injecting a blank saliva sample after the calibration standard of MPA at the upper limit of quantification (high QC) in five replicates.

The stability of the analyte in saliva samples was assessed using six different conditions with independently prepared saliva samples containing MPA concentrations in a matrix equivalent to high and low QC concentrations. For each condition, five replicates of each concentration were tested to stimulate possible storage conditions encountered from saliva collection sample storage during analysis. The first condition involved subjecting the samples to six months of freezing at − 80 °C. The second condition involved three cycles of freezing (at least 12 h at − 80 °C) and thawing at room temperature (20 °C) before analysis. For the third and fourth conditions, the samples were left at 4 °C and at room temperature, respectively, and re-analyzed after 4 h and 2 h, respectively. The fifth condition was used to check the stability of processed samples in an HPLC autosampler at room temperature for 2 h. For all five conditions, 100 µL of saliva spiked with MPA, 50 µL of methanol and 50 µL of IS were mixed and evaporated at 45 °C for 2 h. The dry extract was dissolved in the mobile phase, and after centrifuging twice the supernatant was analyzed using HPLC-FLD method. Freshly prepared IS solution was always used. Lastly, the stability of the dry extract was checked by evaporating the samples to dryness at 45 °C for 2 h, leaving them at 4 °C for 20 h, and subsequently dissolving the dry extract in the mobile phase, centrifuging twice, and analyzing it with HPLC method. The results were compared with the nominal concentrations values.

To assess whether the plain cotton swab from Salivette^®^ absorbs MPA, we measured the recovery of MPA from Salivette^®^. First, Salivette^®^ swabs were spiked with low and high QC concentrations of MPA in previously collected saliva solutions. Second, the samples were incubated for 1 min at 37 °C to simulate the time of saliva collection. Third, the samples underwent the same procedures as the samples for stability tests described above for preparing analytical samples for HPLC analysis (evaporation to dryness and dissolution in the mobile phase). Five replicates of each concentration were analyzed, and recovery was assessed with reference to the nominal concentration of MPA, expressed as a percentage.

### In vivo application

The analytical method was used to determine MPA concentrations in saliva samples collected from two pediatric patients (a boy, child No. 1, and a girl, child No. 2, both aged 12) with nephrotic syndrome who were being treated with MMF at the Department of Pediatric Nephrology and Hypertension at Poznan University of Medical Sciences (Poznań, Poland). Both children have been treated with the same MMF dose for at least one month prior to saliva and blood collection. The study was approved by the Ethical Committee at Poznan University of Medical Sciences (Decisions numbers 773/21 and 808/22). Saliva samples for sMPA determination and plasma samples for tMPA and fMPA determination were collected at the same time at the following time points: before the administration of the next MMF dose, and subsequently 1, 2, 3, 4, 6, 9, 12 h afterward. Saliva was collected using Salivette^®^ devices, with being kept in the mouth for 1 min and immediately centrifuged according to the manufacturer's instructions. Plasma samples were obtained by centrifuging the whole blood for 10 min at 1620 g. Both the saliva and plasma samples were stored at − 80 °C until analysis. For each child, pharmacokinetic parameters such as the concentration before the next dose administration (C_trough_), maximum concentration (C_max_), time to reach maximum concentration (t_max_), secondary C_max_ (C_max2_), time to reach C_max2_ (t_max2_), and AUC_0-12_ using the trapezoid method were calculated. Additionally, sMPA and fMPA free fraction and % of protein bound were also calculated.

## Results

### Chromatography results

**Fig. 1:**
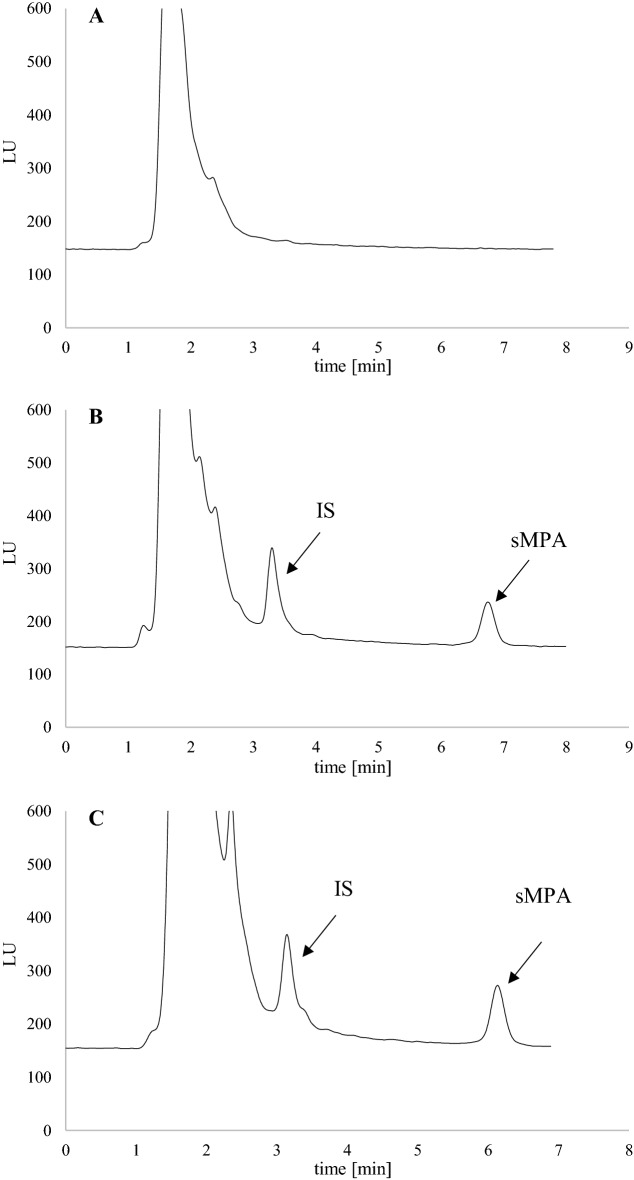
The high-performance liquid chromatography (HPLC) chromatograms of **A** blank saliva, **B** saliva spiked with mycophenolic acid (sMPA; 100 ng/mL in the matrix), and internal standard (IS, 1000 ng/mL in matrix), **C** saliva collected from 12-year old girl treated with mycophenolate mofetil (MMF) 1 h after drug administration

The elaborated HPLC-FLD method was highly selective as no interfering peaks from endogenous substances, or the analyzed drugs were observed. Moreover, the analysis of clinical samples did not show any interfering peaks. The representative chromatograms are presented in Fig. 1 (blank, spiked, child). The average retention time for MPA and IS was 6.7 and 3.3 min, respectively.

### Validation results

The method showed linearity within the 5–2000 ng/mL range, and accuracy and precision met the acceptance criteria for both within-run and between-run analyses (Table 1). The LLOQ for sMPA was 5 ng/mL. The intercept value of the mean standard curve was not significant (t-Student test, α = 0.05).

**Table 1:** Equation of calibration curve, precision and accuracy of the method for MPA in saliva

Calibration curve			Nominal concentration of MPA (ng/mL)	Within-run (n = 5)			Between-run (n = 5)		
Range (ng/mL)	Equation	Correlation coefficient		Mean assayed value (ng/mL)	Accuracy (%)	Precision (CV%)	Mean assayed value (ng/mL)	Accuracy (%)	Precision (CV%)
5–2000	P_MPA_/P_IS_ = 9.0336 × C_MPA_	0.9999	2000	1836	8.2	6.1	2011	0.5	2.5
			1000	1074	7.4	6.0	982	1.8	9.8
			10	8.5	13.6	5.3	8.9	10.8	2.7
			5 (LLOQ)	5.4	7.9	11.8	4.7	6.2	15.2

*C*_*MPA*_ concentration of mycophenolic acid; * CV%* coefficient of variation; * IS* internal standard; * LLOQ* lower limit of quantitation; * MPA* mycophenolic acid; * P*_*MPA*_ peak area of mycophenolic acid;* P*_*IS*_ peak area of internal standard

The stability tests demonstrated that MPA was stable in saliva samples after 6 months of storage at − 80 °C, with a mean accuracy of 6.22% and 12.81% for high and low QC, respectively. MPA was also stable after three freeze–thaw cycles, as the accuracy of the re-analyzed samples ranged from 1.9 to 7.7% of the nominal concentration. MPA in saliva samples was stable if stored at room temperature for 2 h, with a mean accuracy of 2.2% and 5.0% for low and high QC, respectively, and at 4 °C for 4 h, with a mean accuracy of 5.4% and 2.8% for low and high QC, respectively. The stability of MPA in dry extract stored at 4 °C for 20 h was also confirmed, with a mean accuracy of 8.2% and 13.6% for high and low QC, respectively. MPA was stable for 4 h in HPLC vials kept at room temperature in an autosampler, with mean accuracy of 12.0% and 2.9% for low and high QC, respectively.

The recovery of MPA from the cotton swabs was 94% and 105% for high and low QC, respectively.

### Application

**Fig. 2:**
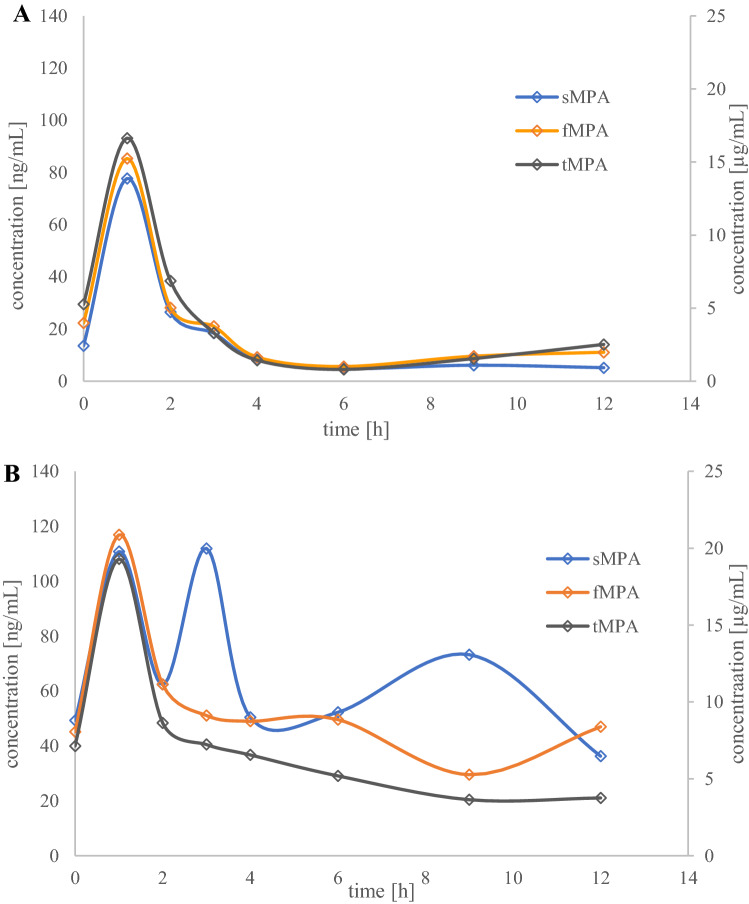
The pharmacokinetic profiles of saliva mycophenolic acid (sMPA), plasma-free mycophenolic acid (fMPA) and plasma total mycophenolic acid (tMPA) for two children (**A** child No. 1; **B** child No. 2) treated with mycophenolate mofetil (MMF)

The method was successfully used to determine sMPA concentrations in two children with nephrotic syndrome who were treated with MMF. The sMPA concentrations ranged from 5 to 112 ng/mL, while tMPA and fMPA in plasma varied from 0.86 to 13.53 µg/mL and 3.4 to 60.5 ng/mL, respectively. The values for sMPA, fMPA, and tMPA pharmacokinetic parameters in child No. 1 and child No. 2 are presented in Table 2. We did not observe a C_max2_ for tMPA, fMPA, or sMPA in child No. 1. In child No. 2, there was a C_max2_ for sMPA, but not for tMPA or fMPA. The pharmacokinetic profiles for both children, including sMPA and fMPA, and tMPA, are presented in Fig. 2.

**Table 2:** The concentrations and pharmacokinetic parameters of sMPA, fMPA and tMPA

Parameter	sMPA		fMPA		tMPA	
	Child No1	Child No2	Child No1	Child No2	Child No1	Child No2
C_trough_ (ng/mL)	14	49	22	45	5.3	7.1
C_max_ (ng/mL)	78.0	111.0	85.0	117.0	16.6	19.3
t_max_ (h)	1	1	1	1	1	1
C_max2_ (ng/mL)	–	73.0	–	47.0	–	–
t_max2_ (h)	–	9	–	12	–	–
AUC_0–12_ (ng h/mL)	180	790	219	609	42.0	78.1
free fraction	0.43	1.01	0.52	0.78	–	–
% of protein bound	99.57	98.99	99.48	99.22		

AUC_0–12_, area under the concentration–time curve from 0 to 12 h; C_trough_, the concentration before the next dose administration; C_max_, maximum concentration; C_max2_, the second maximum concentration; fMPA, free mycophenolic acid; sMPA, saliva mycophenolic acid; t_max_, time of maximum concentration; t_max2_, time of the second maximum concentration; tMPA, total mycophenolic acid

## Discussion

Since sMPA is a free form of the drug not bound to proteins, the analytical method used for its determination must be capable of detecting small concentrations in small volumes and meet the accuracy and precision requirements [25]. Compared to LC–MS/MS analysis, our HPLC-FLD method offers several advantages. It is a more cost-effective option, which is particularly beneficial for research institutions or smaller laboratories with limited budgets. In addition, HPLC equipment is more widely available and easier to handle, with a wealth of knowledge and expertise available for its operation and maintenance. While both techniques are powerful analytical tools, HPLC-FLD provides a more accessible and simpler option.

As the values of tMPA pharmacokinetics were shown to be higher in children with nephrotic syndrome [3–5], we analyzed the linearity of the method over a wide range and with a higher upper limit (5–2000 ng/mL) than in other literature studies. Our method meets the requirements for within-run and between-run accuracy and precision, with the sMPA LLOQ being 5 ng/mL, which is the same as in one study in the literature [22]. Generally, LC–MS/MS methods are more sensitive and show lower LLOQ, with values of 2.5 ng/mL [19], 2 ng/mL [16], 1.6 ng/mL [20], and 1 ng/mL [15]. However, one study reported a higher LLOQ of 150 ng/mL [8]. Our results contradict the study by Shen et al. [16], in which the authors stated that the low sensitivity of HPLC methods (the lowest LLOQ was 5 ng/mL [26] in the studies cited by Shen et al.) makes them unsuitable for fMPA analysis. We used HPLC-FLD methods for both saliva and fMPA determination. In the future, a large number of biological samples should be analyzed to confirm the suitability of the LLOQ in our study, as well as the necessity of a 2000 ng/mL upper limit.

The IS, levofloxacin, used in our study, is contraindicated for use in children and adolescents. Therefore, while our method can be applied to other groups of pediatric patients treated with MMF, it cannot be used for adults treated concomitantly with MMF and levofloxacin. The method is selective, as we did not observe any interfering peaks in saliva samples from healthy volunteers, and no carry-over effect was observed. Additionally, we found that drugs such as paracetamol, voriconazole, itraconazole, ketoconazole, and amlodipine did not interfere with MPA and IS determination. Thus, the method may be applied to children after transplantation who are co-treated with antifungal agents. Children with nephrotic syndrome are frequently treated with steroids in combination with MMF. In our study, we assumed that steroids do not interfere with MPA because their fluorescence is enhanced only after certain treatments of the biological samples, such as derivatization [27], which was not performed in any of the steps in our methodology.

The retention time of MPA was generally longer in our study (6.7 min) than in other studies, which reported times of 3.7 min [16], 5.0 min [19], or 1.0 min [28]). Shorter analysis times are desirable for economic reasons as they save time and reagents [28]. However, the overall analysis time for a single sample with our HPLC-FLD method is still shorter than 10 min.

According to the literature [29], saliva samples should be refrigerated at 4 °C until further processing, which should occur within 3–6 h after collection. Ferreira et al. [22] demonstrated 8-h stability of saliva samples at room temperature. In our study, we found that MPA was stable in saliva samples for up to 2 h at room temperature, as the samples were processed immediately after collection, and there was no need to extend the time for stability testing. We also found that MPA was stable in saliva samples stored at − 80 °C for six months, which is consistent with the Ferreira et al. study [22], which demonstrated the stability of sMPA under the same conditions for 3 months, and its degradation after 3-month storage at -20 °C (short-term stability). In contrast, Brooks et al. [15] showed the stability of MPA in both saliva and plasma matrices for at least 2 years when stored at − 20 °C. Unlike Ferreira et al. [22], we demonstrated the freeze–thaw stability after three cycles and shorter stability of the samples in the autosampler (4 h vs. 12 h) at room temperature. According to Shen et al. [16], the samples stored at 4 °C in the autosampler were stable for up to 15 h. In our study, we also demonstrated that MPA in saliva remains stable for at least 4 h if stored at 4 °C, and the stability of MPA in dry extract stored at 4 °C is at least 20 h.

There are several methods for collecting saliva, including spitting, suctioning, swabbing, draining, and using absorbent materials [15]. In our study, we used Salivette^®^ devices, as in other studies [15, 20–22], to collect stimulated saliva using a cotton swab. Passive drool [19], which does not stimulate saliva production, is also possible for sMPA analysis. It is important to note that the method of saliva collection can affect the results, particularly if it stimulates saliva production, as this may impact the drug concentration, saliva analysis, and composition [15]. Additionally, the swab itself may absorb certain substances, including drugs. However, our recovery results showed that the cotton swab we used for collecting samples from healthy volunteers and children did not absorb MPA. We kept the swab in the mouth for 1 min, following the manufacturer’s instructions, and immediately centrifuged the Salivette^®^ tubes during recovery analysis to mimic the same conditions. It should be noted that MPA can be absorbed by the cotton swab if the samples are not immediately centrifuged, so further analysis is required to determine the appropriate time and temperature for sample storage before centrifugation.

Brooks et al. [15] observed significant variability in sMPA concentrations in renal transplant recipients treated with enteric-coated mycophenolate sodium (EC-MPS), but overall, the sMPA concentrations were similar to previously reported values. In our study, we successfully applied the method to determine sMPA concentrations in two children with nephrotic syndrome to test its feasibility. The sMPA in samples collected 2 h after MMF administration were 27 and 62 ng/mL for child No. 1 and child No. 2, respectively, whereas, in another study, the concentration in a renal pediatric patient was 57 ng/mL [20]. The sMPA AUC_0-12_ for the children in our study was 180 ng h/mL for child No. 1 and 790 ng h/mL for child No. 2. In Brooks et al. study [15], the median sMPA AUC was 216.2 ng h/mL with a range of 126.6–592.6 ng h/mL. The range of sMPA in our study was 5–112 ng/L, whereas other authors reported a range of 1–819 ng/mL [15], 2.6–220.4 ng/mL with a mean sMPA of 31.4 ng/mL [19], and 2.6–220.4 ng/mL with a mean sMPA of 31.4 ± 32.3 ng/mL in adult transplant recipients [19]. As our study only included two children, it is not possible to draw definitive conclusions or compare the results with the literature.

Several studies have confirmed the correlation between plasma and sMPA [8, 16, 19, 20]. It must be emphasized that all the patients received MMF, which is one of two MPA formulations available. However, more recent studies have suggested that sMPA concentrations poorly reflect its plasma concentrations [15, 21]. Cossart et al. [21], reported that in transplant recipients, MPA secretion into saliva may be affected by several factors, such as concomitant medications, which may affect saliva flow rate, mouth pH and saliva enzymes, contamination from food intake; blood in the saliva; and patient disease states. The method of saliva collection may also affect the findings. In patients treated with EC-MPS, sMPA concentrations did not correlate with fMPA and tMPA plasma concentrations, and early post-transplant period, concomitant medications, and disease state may have affected the transfer of MPA into the oral fluid. Generally, EC-MPS is associated with significant pharmacokinetic variability, and the formulation should not directly affect the relationship between plasma and sMPA concentrations. However, the authors concluded that in the case of EC-MPS, the measurement of sMPA concentrations cannot replace plasma measurement for MPA TDM [15]. In our study, we found that in the first child, the correlations of sMPA-tMPA and sMPA-fMPA concentrations were > 0.900 (0.9802 and 0.9943, respectively), whereas these results were lower for the second child (0.6420 and 0.5600 for sMPA-tMPA and sMPA-fMPA, respectively). Shen et al. [16] found that in healthy volunteers and renal transplant recipients, these correlations were above 0.800 and concluded that saliva may be an alternative matrix to plasma tMPA and fMPA monitoring for renal transplant patients.

One limitation of our study is the limited number of samples collected within 12 h after MMF administration, particularly around sMPA C_max_, due to ethical considerations as the study group involved children. As multiple samples were collected within 12 h from pediatric patients, the children were not restricted from flossing or brushing their teeth, nor from eating or drinking 30 min prior to saliva collection. It is important to note that, in light of the Covid-19 pandemic, further studies should investigate the effect of various inactivation treatments for Covid-19 on sMPA concentrations, as saliva contains the SARS-CoV-2 virus and other potential contaminants. Therefore, special attention must be paid to virus inactivation methods in this biological matrix, such as physical and chemical methods, including heat, UV radiation, and the use of different chemicals (e.g., detergents and trizol) [30, 31]. It has been demonstrated that temperatures above 50 °C can effectively inactivate the virus, and further studies are needed to determine whether MPA remains stable under such conditions [30]. The same issue applies to UV light exposure and chemical procedures. Recently, it was shown that Triton X-100 and NP-40 did not affect enzymes present in saliva but increased saliva levels of cortisol, triglycerides and glucose [32]. In our study, we implemented safety measures such as the use of protective gloves and glasses and disinfectant liquid to clean the test tubes and working surfaces.

To conclude, the established HPLC-FLD method for sMPA determination is specific and selective and meets the validation requirements for the analytic methods. This method may be used in children with nephrotic syndrome as well as it is possible to apply it in other groups of pediatric patients e.g., after transplantation. HPLC-FLD method was not applied for sMPA determination so far, but it may be successfully used instead of LC–MS/MS methods due to its cost-effectiveness, wide availability, and easier handling. Further studies are needed to investigate whether sMPA concentrations can be used as a reliable measure for MPA TDM in children with nephrotic syndrome and if they correlate with tMPA concentrations.

## Data Availability

The datasets generated during and/or analyzed during the current study are available from the corresponding author upon reasonable request.

## Abbreviations

AUC_0–12_: Area under the concentration–time from 0 to 12 h curve
C_max_: Maximum concentration
C_max2_: Secondary maximum concentration
CV%: Coefficient of variation
EC-MPS: Enteric-coated mycophenolate sodium
FLD: Fluorescence detection
fMPA: Free mycophenolic acid
HPLC: High-performance liquid chromatography
IS: Internal standard
LC–MS/MS: Liquid chromatography tandem mass spectrometry
LLOQ: Lower limit of quantification
LSS: Limited sampling strategy
MMF: Mycophenolate mofetil
MPA: Mycophenolic acid
QC: Quality control
SECS: Salivary Excretion Classification System
sMPA: Saliva mycophenolic acid
TBAB: Tetrabutylammonium bromide
TDM: Therapeutic drug monitoring
t_max_: Time to reach maximum concentration
t_max2_: Time to reach secondary maximum concentration
tMPA: Total mycophenolic acid
